# Regulatory sandboxes as an innovative platform for testing Cannabis edibles in Germany

**DOI:** 10.1186/s42238-025-00263-1

**Published:** 2025-03-01

**Authors:** Hana Al Hallaj, Zahraa Barakat

**Affiliations:** 1 Technische Universität Berlin, Building H, Space H4136A, Straße des 17. Juni 135, Berlin, 10623 Germany; 2 Department of Economics, MA in Applied Economics, Lebanese American University, Beirut, P.O. Box: 13-5053, Lebanon

**Keywords:** Medical Cannabis, Innovation, Edibles, Marijuana, Regulatory sandboxes, Germany

## Abstract

This paper explores the effectiveness of using Regulatory Sandboxes (RS) to legalize THC edibles in Germany. While RSs have been extensively studied in sectors like Fintech and Healthtech, their application in introducing novel cannabis products or services remains underexplored. Utilizing Qualitative Comparative Analysis (QCA) across three countries namely Brazil, the state of Arizona and Thailand, we identify potential conditions for successful implementation of RS in the cannabis industry. Consequently, we propose the establishment of a tailored RS in Germany for cannabis edibles, aiming to foster innovation and drive revenue within the cannabis sector. The paper introduces a novel concept and paves the way for more research in the fields of RS and cannabis.

## Introduction

This paper discusses the concept and implementation of RSs particularly in the cannabis industry. Within the European Union (EU) and Germany, RSs serve as controlled environments for testing and innovating new products and services under regulatory oversight. While they initially gained prominence in the financial industry, their scope has expanded to include diverse sectors such as healthcare, transportation, and energy.

The German government’s RS strategy, aims to evaluate innovations and regulations, improve the legal environment, and encourage digital innovation. The strategy involves experimentation clauses, a flexible regulatory framework, network infrastructure improvement, and support mechanisms for pilot projects. It suggests that real laboratories, governed by the Real Laboratories Act^1^, can serve as comprehensive solutions, offering advisory services, knowledge transfer, regulatory feedback, and scalability throughout the evaluation process. This approach facilitates transparency and accessibility to progress, allowing legislators to gain insights for legal modifications.

In this research, the shift towards edible cannabis products in the US is explored, driven by factors such as perceived safety, health considerations, and convenience, and their popularity is reflected in market data from California and Colorado. The literature review delves into the complexities of cannabis consumption, including oral cannabinoids and the challenges of determining optimal dosage for both recreational and medicinal use. It also addresses the risks associated with cannabis, particularly in the context of edibles, emphasizing the importance of stringent regulations, user education, and quality control. The review on edibles concludes by proposing collaborations between the cannabis industry and established fields like food and pharmaceuticals to enhance the development and safety of cannabis edibles and beverages, acknowledging the need for continued improvement and innovation in this rapidly expanding industry.

In light of Germany’s progress towards cannabis legalization and the enactment of the Cannabis Act in April 2024, there exists a logical and timely opportunity to test cannabis edibles within a RS framework. This strategy is consistent with the country’s progressive position on cannabis usage and offers a controlled setting for testing the safety, effectiveness, and market readiness of cannabis edibles.

The concept of an RS has been widely recognized as an effective tool for innovation while mitigating risks. As such, by establishing a RS, German authorities may collect critical data on consumption patterns, health effects, and possible societal ramifications, ensuring that the introduction of cannabis edibles is handled responsibly and in accordance with public health goals and safety regulations.

This study aims to answer the following question: Are RSs effective in testing cannabis edibles in Germany? To answer this question, the paper will focus on the following framework:

- Highlighting the uses, advantages, and disadvantages of RS.
- Understanding the regulatory and market conditions through identifying the essential factors that would influence the successful implementation of RS in the context of cannabis edibles.
- Assessing the feasibility of RS for Cannabis Edibles in Germany by examining other countries’ experiences in the cannabis sector.

This analysis will contribute to the formulation of informed regulatory measures that promote the safe and responsible growth of the market. The section that follows is a literature review of RS, RS in Germany and the edibles shift. The following section describes three cannabis case studies whereby RS are implemented or were planned to be implemented to address the introduction of a new cannabis law or regulation into the country. After a QCA is conducted, the study concludes with the recommendation of using RS as a tool to test the possible introduction of cannabis edibles in Germany. This study is qualitative in nature and is limited by the lack of available data and resources. More research and analysis are required in the future to tackle this subject in more depth.

## Literature Review

### Introduction to RSs

#### RS Overview

RSs, though lacking a universally agreed-upon definition, are predominantly recognized as specialized platforms. These platforms enable organizations to test new products and business models under the structured oversight of regulatory bodies, but only for a limited time (Rinnge and Rouf 2020). Their twin objectives are to foster business innovation through practical testing in real-world scenarios and to assist in the development of legal frameworks that effectively guide and support these entrepreneurial activities (Ranchordas 2021a; Leimüller and Wasserbacher-Schwarzer 2020; EIPA 2021).

Although their main application lies in the financial industry, specifically for fintech advancements such as digital wallets and IDs, they have progressively emerged as a crucial tool in the EU for steering the progress and execution of state-of-the-art technologies, such as AI and blockchain, in various sectors (Ringe and Ruof 2018). The RS encompasses self-driving vehicle technology in transportation, smart meter applications in energy, and breakthroughs in early illness identification and assessing antidepressant therapy responses in the healthcare sector among others. (World Bank 2020).

The initiation of a RS can occur either through innovators identifying regulatory challenges (the bottom-up approach) or through regulatory authorities setting terms and inviting proposals (the top-down approach), offering a flexible approach to integrating emerging technologies within regulated frameworks (Council of the European Union 2023).

Within the realm of regulatory experimentation, certain phrases are often used interchangeably, however, they possess unique definitions. For example, throughout history, experimental approaches have played a crucial role in facilitating the integration of new technology into society. The terms “experimental legal regimes” or “experimental regulation” refer to the practice of conducting trials with laws and regulations (Ranchordas 2021a). These trials sometimes include temporary departures from established standards in order to assess the effectiveness of various legal frameworks in certain areas (Heldeweg 2017). “Experimental sandboxes,” which are often referred to as “RSs”, are a newer and more specific version of these experimental systems. They include cooperation between the public and private sectors (Lim and Low 2019).

#### Types of RS

Within RS environments, there are four types that may be distinguished, each serving certain objectives. Policy-focused sandboxes are used to examine and analyze certain policies or legislation. Innovation- or product-focused sandboxes seek to promote innovation by reducing the obstacles to entering the regulated market and allowing companies to assess the commercial viability of new models. Thematic sandboxes focus on certain topics to accelerate the implementation of particular technologies or regulations or to boost the development of specific subsectors or goods for targeted populations. Finally, cross-border sandboxes enable businesses to expand internationally, foster collaboration among regulators, and strive to reduce regulatory discrepancies (World Bank 2020).

#### Potential drawbacks of RSs

As a response to the challenges presented by the swift advancement of emergent technologies, Johnson (2023) proposes that decision-makers adopt a prudent strategy including ensuring consistent resources when considering RSs, recognizing that they may not always provide the best solution to regulatory concerns in various sectors. He presents a comprehensive framework rooted in regulatory literature, combining five fundamental components: process-oriented regulation, outcomes-driven orientation, regulated discretion, approval regulation, and structured communication between regulators and stakeholders.

For instance, trust is a crucial component of effective cooperation and communication in RSs, so it is important to build long-lasting trust with regulators and third-party actors (Peake and Forsyth 2021). Analysis of the evolving relationships between regulators and regulatees provides valuable insights into sandbox performance and conditions for enhanced compliance, as opposed to exclusively focusing on regulatory design. Public or third-party participation in the regulatory framework can increase the accountability and robustness of a program; however, effective inclusion strategies must be developed and empirically evaluated (Johnson 2023).

As per Sherkow 2022; the potential downside to implementing RSs is foremost the significant risk of consumer harm. Many formal regulatory programs aim to protect consumers from harm before the introduction of potentially unsafe technologies. The widespread deployment of technologies that are less than safe or functional may erode public trust in the field regulated by the sandbox. There is also concern that having parallel regulatory tracks—one slow, deliberate, and punitive when errors occur, and another quick, liberal, and forgiving—might lead to a preference for the less restrictive model, creating a slippery slope.

Finally, the way regulators respond to actual or perceived political pressures within the flexible sandbox framework can have a significant impact on policy outcomes (Haines 2011).

#### RS implementation

A RS process consists of a set of separate and systematic steps meant to permit successful testing and evaluation of new initiatives (Organisation for Economic Co-operation and Development 2023). These stages include the following steps:

i. Application and Selection: During this first round, enterprises apply to join the RS based on predetermined criteria.
ii. Contact and Planning: Once the enterprises are chosen, a comprehensive testing plan is prepared (if the participants are already confirmed) which specifies the components of the enterprises that will be examined, developed, or altered within the RS.
iii. Monitoring and Consultation: As the endeavors go through the testing phase, the RS provider actively observes the procedures and consults as needed. This stage is crucial for resolving any new difficulties and ensuring that the set testing strategy is followed.
iv. Evaluation and Reporting: The RS provider evaluates the results after a predetermined period, frequently in collaboration with the participating businesses. This testing report throws light on the results, issues encountered, accomplishments, and possible areas for development discovered throughout the testing phase.
v. Exit and Scaling: In the last phase, participants decide on the scaling and wider implementation of the RS outcomes leveraging on the insights and advances gained throughout the testing period.

### RSs in Germany

The introduction of a RS strategy by the German Federal Ministry for Economic Affairs and Energy commenced in 2018 with the purpose of evaluating innovations and regulations. (BMWi 2018). Since 2019, the Coordinating Office for RSs at the Federal Ministry for Economic Affairs and Climate Action (BMWK) has been implementing a plan consisting of three pillars for RSs. The first pillar consists of legal possibilities, such as establishing consistent standards and including experimentation provisions. The second pillar encompasses the exchange of information and networking via services such as interministerial working groups, handbooks, and networks. Facilitate and provide support for initiatives such as the RSs Innovation Prize and pilot project help from the third pillar (BMWK 2022).

The significance of RSs in positioning Germany as a hub of innovation is growing. These platforms provide the means for innovative organizations, governmental entities, and researchers to test concepts that were inconceivable just a few years ago (BMWK 2023).

In terms of constructing experimental clauses, the German government identified five crucial steps. This methodology, outlined in the “New Flexibility for Innovation” guide, presents a comprehensive approach to developing these clauses, ensuring their effective integration within legal frameworks for fostering innovation (BMWi 2019).

In living laboratory evaluations, the obligations of state protection and the industrial property rights of innovators must coexist (Fahey et al. 2022). From these laboratories, legislators can gain knowledge and make legal modifications, thereby assuring transparency and accessibility to progress. On November 13, 2024, the Federal Cabinet approved the draft law to improve the framework conditions for testing innovations in real-world laboratories and to promote regulatory learning (Real-world Laboratories Act - ReallaboreG). The draft law provides for the first time legal regulations for definitions of real-world laboratories, experimental clauses and regulatory learning, Reallabore Innovation Portal, innovation-friendly discretionary powers in the approval process and appropriate time limits and extensions of testing in real-world laboratories, regulatory learning in real-world laboratories, and a reporting obligation of the BMWK to the Bundestag (BMKW 2024).

Table 1 summarizes the main differences between the German and EU notion of sandboxes:

**Table 1:** German and EU Sandbox differences

German Reallabore (Sandbox)	EU Sandbox
Hub for Innovation: Generally regarded as the primary center for promoting technical innovation and progress, especially in certain industries.	Discrete Legal Experimentation: focuses on the precise examination and improvement of certain legislative provisions, enabling focused regulatory experimentation.
Improving Legal Framework: The goal is to improve the legal environment to better foster innovation and the exchange of knowledge across companies and sectors, while maintaining a controlled approach to risk management.	Extensive Legal Acknowledgement: highlights how crucial it is to widely acknowledge and apply legislative reforms based on experimental results, while encouraging more exploratory approaches to innovation and risk.
Sector-Specific Focus: Focuses on key sectors, offering support for the standardization and alignment of industry practices and frameworks	Comprehensive and Diverse: encompasses a larger number of businesses and sectors and allows for a wider array of experimental strategies and regulatory modifications.
Experimental Flexibility: Experimentation provisions are used to provide flexibility to the regulatory framework, enabling changes based on empirical testing.	Dynamic Adaptability: Extremely adaptable to novel challenges and advancements, and highly sensitive to the rapid evolution of developing technology.
Policy and Legal Strengthening: Focuses on modifying and strengthening existing policies and legal processes to ensure they remain conducive to innovation.	Regulatory Evolution: promotes the testing, learning, and adaptation of regulatory frameworks to changing legal and technological contexts on an ongoing basis.

Source: Authors’ summary based on BMWK and EC (European Commission) implementation of Sandboxes

### Introduction to edibles

Cannabis, acknowledged as the most often utilized illicit substance in the United States, is extensively consumed by a considerable proportion of both adults and young individuals (Hasin et al. 2015; Johnston et al. 2016). The surge in its popularity can be attributed, in part, to the decreasing perception of risk (Johnston et al. 2016; Miech et al. 2015; Okaneku et al. 2015), as well as the growing trend of legalizing marijuana for both medical and recreational use in some jurisdictions (Cerda et al. 2012).

As of February 2024, 47 states, the District of Columbia, and 3 territories (Guam, Puerto Rico, U.S. Virgin Islands) allow for the use of cannabis for medical purposes. 38 states, the District of Columbia, and 3 territories allow for the use of cannabis for medical purposes through comprehensive programs. 14 states and 2 territories have a comprehensive medical-only program. 9 states have medical programs that only allow for the use of CBD/low-THC products for qualifying medical condition(s) as defined by the state. Comprehensive medical programs, in this context, refer to programs that allow for the use of cannabis products beyond CBD/low THC for medical purposes as defined by the state or territory. 24 states, the District of Columbia, and 2 territories (Guam and North Mariana Islands) allow for the use of cannabis for non-medical adult purposes.^2^ Many of these jurisdictions allow cannabis-infused edibles to be manufactured, sold, and consumed. In a ground-breaking development in the same year, the US Department of Justice proposed rescheduling cannabis as a controlled narcotic.

Medicinal use of cannabis was legalized nationwide under conditions outlined in the Marijuana for Medical Purposes Regulations issued by Health Canada on 30 July 2001. In 2018, the Canadian Parliament approved a final, reconciled version of Bill C-45 enabling marijuana to be legal for adults. As of 2019, the government of Canada introduced regulations pertaining to the legalization of cannabis edibles and beverages (Government of Canada 2001).

While over 20 European countries have introduced medical cannabis legislation, recreational legalisation remains mixed. Germany is leading with its partial legalisation, and other countries are exploring non-profit models and pilot programmes for navigating EU and UN regulations (https://practiceguides.chambers.com/practice-guides/medical-cannabis-cannabinoid-regulation-2024). Germany legalized cannabis for medical use in 2017. Following a controversial national debate about the pros and cons of allowing easier access to cannabis, Germany’s house of parliament voted to legalize cannabis in early 2024 for limited recreational use. Under the new Cannabis Act, adults can cultivate up to three plants for private consumption and be allowed to possess 50 g at one time at home, and 25 g in public, starting from April 1. From July 1, cannabis will be available in licensed not-for-profit clubs with no more than 500 members – all of whom would have to be adults. Only club members would be allowed to consume their output. The German government said that cannabis would remain illegal for minors and highly restricted for young adults, adding that consuming the drug near schools and playgrounds would be illegal (Bundesgesundheitsministerium, 2024). Although there was no mention of an experimental clause, the German government opted a 2-pillar approach to legalize adult-use cannabis gradually. The law has made no reference to cannabis edibles but focusses on cannabis flos or cannabis dried flower.

Recreational use aims to get a “high” or change one’s mental state without a medical reason, while medicinal use requires a prescription from a doctor. These two categories typically overlap since recreational users may self-medicate with cannabis and medicinal users may have used it recreationally. The regulatory and taxation differences between medicinal and retail cannabis markets are crucial. (MacCoun and Mello 2015; Monte et al. 2015).

Cannabis consumption varies in form, including smoking, oils, and edibles, each offering a different experience and effect. While smoking is a common method, consumers were more inclined to opt for edibles as an alternative to cannabis inhalation. This change was triggered by several factors. First, edible cannabis products are perceived by consumers as a secure and healthier alternative to smoking or vaporization. Second, certain physicians who prescribe cannabis to patients in Canada suggest oils and capsules as effective substitutes to smoking. In addition, consumables are more easily ingested and do not require any preparation in contrast to smoking. Finally, edibles are also more convenient as they can be easily utilized in outdoor settings such as social gatherings or events (Blake and Nahtigal 2019).

Detailed figures for these product categories, including units sold and average retail prices, are presented in Table 2 below (Blake and Nahtigal 2019).

**Table 2:** Market data for consumable and beverages in California and Colorado

Product category	Sales in 2018	Units sold	Average retail price/unit
Dried flower	$1.6 billion	250.87 million grams	$6.38
Ingestible	$625.99 million	31.96 million	$19.59
Beverages	$35.79 million	2 million	$14.90

Source: Blake and Nahtigal 2019, p.28

Edibles offer advantages such as a longer duration of action and analgesic effect, which are particularly beneficial for medicinal cannabis users with chronic conditions (Huestis 2007). While gaining popularity as a treatment option, the impact of edibles, particularly on brain development and mental health, is a significant concern (Volkow et al. 2014).

In light of these possible risks, ascertaining the optimal dosage of 9-THC for recreational and medicinal use continues to present an immense challenge. Misreading the labels can cause people to take too much (Hudak et al. 2015), and because everyone reacts differently to 9-THC, it’s hard to set standard dosage equivalents (Groteenhermen 2001). Regulatory initiatives, such as specified serving sizes and slow titration protocols, attempt to reduce the danger of accidental overdose (Grothenhermen 2003).

Because of federal prohibitions on cannabis in the US, states vary in their edible labeling requirements. For example: nutritional information labels, details regarding quality control testing, child-resistant packaging, consistent distribution of Δ9-THC, and comprehensive inventory management from cultivation to sale are obligatory in some states. Moreover, the production of potentially child-appealing consumables, including candies, is explicitly forbidden in the following states: Washington, Alaska, and Oregon (Barrus 2016).

Increased unintentional exposures and related calls to poison control centers in decriminalized states (Wang et al. 2014; Cao et al. 2016), as well as an increase in emergency room visits since legalization (Barrus 2016; Kim et al. 2016), highlight the public health concerns associated with edibles. These results highlight the crucial need for stronger regulatory frameworks, standardized procedures, and extensive educational campaigns to promote safe and educated cannabis use, particularly for edibles. However, innovation in analytical testing, formulation development, and manufacturing processes of cannabis edibles might be driven by partnerships with well-established fields such as food or pharmaceuticals. In addition to improving the flavor, dose consistency, and dose homogeneity of consumables and beverages, this collaboration would also leverage the food industry’s knowledge in areas such as sanitation and food safety procedures (Blake and Nahtigal 2019).

## Methodology

The authors employ a QCA to investigate the efficacy of RSs for introducing new cannabis products such as edibles in Germany. The analysis focuses on three case studies: the state of Arizona, Thailand, and Brazil. The objective is to gain valuable insights from the diverse legislative and operational frameworks of these countries and states.

The authors scraped the internet for any cases of implementing RSs in the Cannabis space. Due to the novelty of the industry and the complexity of the legal landscape globally, only three cases were identified. The search included the keywords of “RS” and “Cannabis”. In all the three cases, the countries or states referred to their testing platform for introducing the cannabis product/ service as RS. For Brazil and Arizona, two secondary sources were identified, and a few media articles were posted on Thailand. This led to performing interviews with key stakeholders in Thailand to ask about the process of the RS and to dig deeper into its implementation.

The study used a dual-pronged approach to gather data on RS applications in the cannabis industry. Busetto et al. 2020 have indicated that the most used methods of qualitative data collection (especially in health research) are document study, observations, semi-structured interviews, and focus groups. The selection of either individual or combined methods should be guided by the research question and a critical assessment of how well the chosen method aligns with the research objectives.

According to Busetto et al. 2020 document study, also known as document analysis, involves the review of written materials by the researcher. These materials can include both personal and non-personal documents such as archives, annual reports, guidelines, policy documents, diaries, and letters. In the state of Arizona and Brazil, secondary sources were reviewed, including scholarly literature, legal documentation, policy analyses, and media reports.

Interviews are particularly valuable for gaining insights into a person’s subjective experiences, opinions, and motivations, rather than merely collecting facts or observing behaviors. The underlying philosophy of qualitative research does not adhere to an objective hierarchy of evidence and methods. Consequently, the choice of methods must be critically evaluated based on the research question and the extent to which the selected method can effectively address it, ensuring an optimal “fit” between question and method. In Thailand, due to the scarcity of existing information, the study relied on conducting interviews with government officials and local industry leaders to understand the operational intricacies, obstacles, and achievements of Thailand’s RS in the cannabis industry.

Arend Lijphart (1971) argued that, whenever possible, one should prefer the statistical or experimental method over the weaker comparative method. However, due to limitations in time, energy, and resources, a thorough comparative analysis of a few cases may be more promising than a superficial statistical analysis of many cases. The comparative method is a broad, general approach rather than a specialized technique. In such situations, the most productive strategy would involve using the comparative method as an initial stage of research before proceeding to statistical analysis particularly when dealing with a small number of cases.

**Fig. 1:**
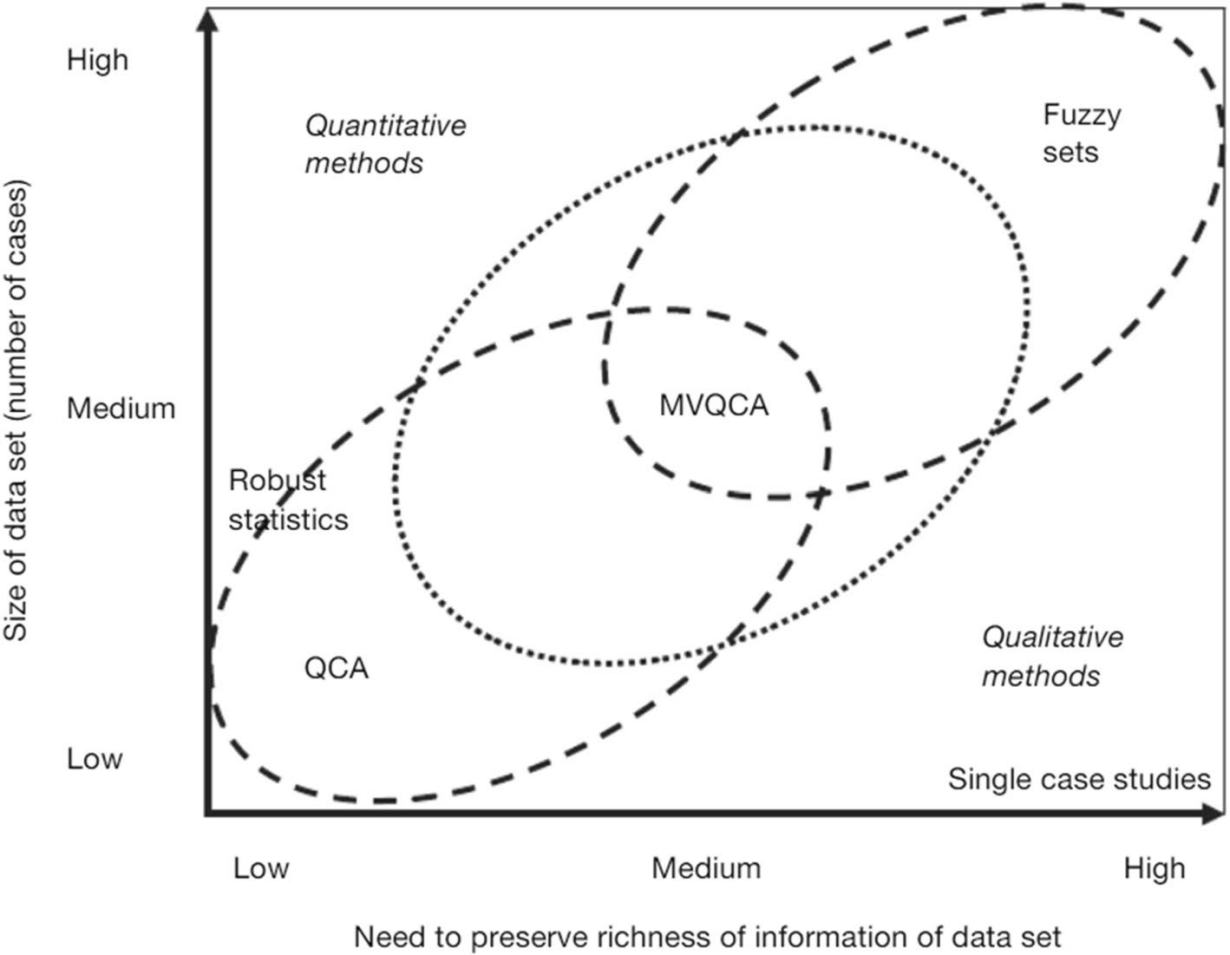
Rihoux (2006)

According to Herrmann and Cronqvist (2005), different comparative techniques are best used in different research situations, following two dimensions. The first dimension is the sheer number of cases – the size of the data set. The second dimension is the necessity to preserve the richness of the data information in the raw data set. Figure 1 summarizes situations in which each one of the three techniques is best used (Rihoux 2006).

Benoit’s illustration in Fig. 1 supports the choice of QCA as an appropriate analysis technique, particularly given the low number of cases and the limited richness of information available. This fit is explained by the two dimensions highlighted above.

Developed in the 1970s by Charles Ragin, QCA is a research methodology designed to analyze complex situations and explain why change occurs in some instances but not in others (Simister and Scholz 2017). The efficacy of QCA in generating meaningful insights and addressing real-world research inquiries has been demonstrated through empirical applications. As a result, the extensive adoption of QCA highlights its utility as a robust analytical instrument that can yield significant understandings of intricate social phenomena (Oana and Schneider 2021).

QCA is particularly valuable for evaluations that seek to understand not just the outcomes of a project or program, but also the processes and reasons behind those outcomes. Consequently, QCA can inform decisions about whether and how projects or programs might be scaled up or replicated (Baptist and Befani 2015). QCA also allows researchers to explore the conditions under which specific policies are effective or ineffective, producing results that are applicable to cases with deterministic characteristics. By modeling complexity, QCA enhances understanding of the relationships between causative factors and outcomes, offering insights into the nuanced dynamics of policy implementation and evaluation. Additionally, QCA facilitates systematic analysis of case study material through a quasi-experimental approach, enabling comparison even with small sample sizes and promoting middle-range generalizations that support cross-national comparisons (Thomann 2020).

QCA has primarily been utilized within the research community or as part of one-off evaluations or impact assessments (Nigel Simister and Vera Scholz,  2017). However, the scoring process for factors in QCA may risk subjectivity. This subjectivity can be problematic when determining key factors in complex scenarios, such as advocacy campaigns, where the impact may take years to materialize and the outcomes are not immediately clear.

In addition, QCA is not able to handle the dynamic aspects of the processes that drive policy outcomes and their changes over time (Thomann 2020). QCA is by nature case-sensitive, implying that outcomes may differ based on the instances considered in the analysis, which can restrict the applicability of findings and create difficulties in drawing broader theoretical implications. Finally, QCA is not particularly useful for determining average effect sizes or precise quantitative correlations between variables. The authors have chosen QCA as the methodology to discuss a novel concept at a strategic level. However, the drawbacks of QCA are acknowledged comprehensively.

Befani (2016) and Thiem (2022) assert that qualitative comparative analysis methodologies require a minimum of three to five cases. Therefore, we can effectively analyze and interpret the causal configurations underlying the cannabis RSs in our study by employing QCA with three cases.

Our study focuses on four main conditions that are expected to influence this outcome significantly, drawing on the findings from the existing body of literature and benefiting from two important studies: Donadelli and van der Heijden (2022), which examines the integration of regulatory practices and distributive policies in developing nations (specifically Brazil), and McCallum and Aziakpono (2023), which offers insightful observations on the conditions required for the effective implementation of RS, with a particular emphasis on South Africa.

- New Industry: Reflecting on the rapid expansion of the FinTech sector, as mentioned by McCallum and Aziakpono (2023), parallels can be drawn to the emerging cannabis market, emphasizing the need for flexible regulatory frameworks that are tailored to the unique characteristics and challenges of each new industry. The significance of adaptability is further reinforced by the examination of regulatory failures in Brazil, which exposes the risks associated with insufficient capacity and understanding in emerging and developing industries (Donadelli and van der Heijden 2022).
- New Cannabis product/service: Introducing new product as seen in the FinTech industry, reflect the critical role of RS in enabling market access and reducing regulatory uncertainties for innovations (McCallum and Aziakpono 2023). Lessons from Brazil regarding the challenges in understanding and effectively regulating new products (Donadelli and van der Heijden 2022) reinforce the importance of RS in offering a structured environment for the safe introduction and regulation of new cannabis products.
- New Policy: According to McCallum and Aziakpono’s (2023) examination, the implementation of new policies through RSs in South Africa is evidence that creative policy instruments can promote industry growth and close regulatory gaps. This approach is consistent with the findings of Donadelli and van der Heijden (2022), who emphasize the importance of implementing innovation-driven and inclusive policy initiatives. These approaches go beyond established regulatory paradigms to address the specific demands and challenges of new sectors. Moreover, Donadelli and van der Heijden (2022) question the traditional view that regulatory agencies should be impartial, apart from policies related to distribution. They claim that in countries with significant socio-economic differences, such as Brazil, it is essential to integrate economic redistribution within regulatory frameworks to achieve success. This approach is particularly pertinent to the cannabis sector since it necessitates laws that consider the socio-economic ramifications and address the needs of many stakeholders.
- Implementation of RSs in Cannabis: By demonstrating how controlled innovation environments can foster expansion while ensuring safety and compliance, McCallum and Aziakpono highlight the advantages of RS in the FinTech industry in South Africa. Simultaneously, on Brazil’s regulatory adaptability, Donadelli and van der Heijden emphasize the vital role of efficient regulatory mechanisms in overcoming barriers such as limited resources and political interference.

This comparative method addresses the “what” while leaving the “how” and “why” to future research endeavors. In addition, the three countries chosen in our study vary significantly from Germany in legal, economic, and social situations. The purpose of selecting these case studies is to give a variety of regulatory experiences that, although not directly comparable, may nonetheless provide useful concepts and cautionary lessons. These insights should be deliberately used, considering Germany’s specific regulatory environment, social attitudes, and legal framework. The purpose is not to recommend a straight implementation of these models, but rather to use their experiences to inspire a tailored approach to Germany’s unique demands.

## Case studies of RSs in Cannabis

### Introducing Cannabis through RS in Brazil

The study on the RS for cannabis in Brazil by Oliveira and Barros de Abreu provides a full examination of creating such a framework for regulating cannabis for therapeutic and palliative reasons. The emphasis in Brazil has been on expediting the regulatory procedure for medicinal cannabis. There were no laws controlling the manufacturing of cannabis-based medications in Brazil until the end of 2019, resulting in exorbitant expenses for patients who had to import these treatments with the license of ANVISA (National Health Surveillance Agency). This circumstance drove numerous patients to file lawsuits against the state to get coverage for these expensive procedures, which had a substantial effect on the Unified Health System (SUS) budget.

As a result, on December 9, 2019, ANVISA passed Collegiate Directory Resolution 327. This resolution effectively served as an RS by setting temporary guidelines for the production and distribution of Cannabidiol-based medications (a new product) in Brazil for a specific period of 5 years as a test period under the supervision of ANVISA in order to aid in developing a legal framework for the product. According to the resolution, these medications may only be administered to individuals who have exhausted all other traditional therapies and are already registered with approved physicians or patients that are under palliative care. Furthermore, these medications must only be offered in pharmacies by registered firms, show the plant’s name, and provide detailed information on consumption and any negative effects. Notably, the resolution prohibited firms authorized to manufacture CBD medications from importing cannabis plants. This preliminary regulatory legislation intended to regulate the manufacturing of cannabis-based medicines, lower treatment costs, promote industry, and benefit patients and the state.

The economic analysis of law technique used in the case study assessed laws from an economic standpoint, with an emphasis on optimizing resources and controlling social interactions. This multidisciplinary approach helped lawmakers and regulators validate the effectiveness of legislation and court judgments. The RS was useful in understanding regulatory repercussions, minimizing barriers to innovation, and avoiding possible damage to the Economic Order, ultimately improving societal welfare.

The research also mentions market flaws that could be obstructing the economic order’s goals as outlined in the Federal Constitution of 1988, such as imperfect competition, externalities, asymmetric knowledge, and inefficient institutions. The RS was depicted as a successful instrument for addressing market failures by providing regulators with actual access to goods and services, ensuring regulatory efficiency and aggressiveness, and limiting state intervention in the economic order. Furthermore, the research emphasizes the significance of free business and competition in fostering social welfare and economic progress, which are fundamental to the Federal Constitution of 1988. The RS promoted these ideals by fostering a secure environment for innovation and aligning laws with the actual demands of society and the economy.

On April 22, 2020, the National Health Surveillance Agency authorized the production of the first cannabis-based drug in Brazil. The regulatory agency finalized the evaluation of the application in 42 days, including the time that the applicant company needed to submit the necessary documents (https://clinregs.niaid.nih.gov/country/brazil.). Although this was still not a definitive and enforceable regulation, but rather rules that will allow previously registered companies to develop and commercialize cannabis- derived medicines within the national territory to previously registered patients, these drugs may or may not be subsequently registered by the National Health Surveillance Agency (ANVISA). The RDC 327/2019 served as a RS providing a reduced environment and preparing the drugs to be analyzed by ANVISA.

In line with the definition of RS in the Literature Review section of this paper, 3 and in the case of Brazil, it was found that the RS contributed to the creation of effective standards. In this tuning fork, the authorization and regulation by the National Health Surveillance Agency (ANVISA) to produce medications for palliative treatments based on Cannabidiol allowed cost reduction, increased free competition, guarantee of free initiative and an increase in social well -being.

### Using RS to introduce Cannabis payments in Arizona

This case study explores the implementation of a payment platform/service specifically designed for the cannabis industry in Arizona. Given the federal illegality of cannabis in the United States, traditional banks were unable to provide financial services to cannabis businesses. Consequently, the State of Arizona initiated a pilot program under the umbrella of a RS to test and evaluate a new financial service tailored for the cannabis sector. Arizona, led by Attorney General Mark Brnovich, launched the FinTech Sandbox program to address the financial challenges in the state’s cannabis industry due to federal banking restrictions. The program aimed to reduce reliance on cash, improve security, and enhance operational efficiency.

The RS allowed financial technology companies working with cannabis companies to test and refine services in a controlled environment, minimizing legal barriers while protecting consumer rights. This enabled businesses to explore new financial strategies without immediate legal constraints, fostering technological improvements in the cannabis sector.

Alta Solutions LLC, a key participant, developed a digital payment network specifically for the cannabis industry. By using geofencing and blockchain, Alta Solutions improved the security of digital transactions, mitigating the risks of cash handling. This approach supported the program’s goals of promoting economic growth and ensuring regulatory compliance (Ringle 2019; Cromley 2019).

The reliance on cash-only transactions had posed significant operational and safety challenges, particularly for medical marijuana dispensaries (Wessel 2021). Alta Solutions’ platform offered a secure and efficient alternative, functioning similarly to digital payment platforms like Venmo, and addressed critical needs such as tax payments.

The sandbox initiative, including Alta’s contributions, represented a broader effort to support innovation in the cannabis sector by encouraging companies to develop financial solutions that met the industry’s complex needs. This collaborative approach aimed to build a financial infrastructure that supported comprehensive growth of the cannabis sector.

### Introducing Cannabis in Thailand

The case study in this section depicts an example of Cannabis legalization that was intended to be introduced through a RS but was blown out abruptly into full legalization before testing. Several interviews were conducted with key stakeholders to understand the process and shed light on the pros and cons of not deciding to utilize a RS as a trial period. All the interviewers concur that legalization in Thailand has been a complicated and stressful process, with substantial alterations in legislation and popular perception.

Since 2018, Thailand has permitted the use of medical marijuana in an effort to improve public health and well-being. In addition, with the implementation of decriminalization in 2022, the cultivation, trade, and medical use of marijuana and hemp products, as well as the extraction of any portion of the plant, ceased to be illegal activities. (PR Thai Government 2022). The decriminalization of cannabis was perceived as a forward-thinking measure aimed at stimulating agricultural development and boosting the country’s tourism sector (Olarn 2024).

Subsequently, Thailand witnessed a cannabis boom with the expansion of thousands of cannabis-themed businesses throughout the country. Even cities such as Bangkok and Chiang Mai hosted cannabis festivals, which attracted both tourists and enthusiasts. Advocates for the legalization of cannabis contended that it yielded advantages for a multitude of societal segments, such as small business owners, cannabis industry employees, and cultivators.

In fact, the Thai government was in the process of introducing RSs for cannabis use in 2022 (Belaws and The President of Phuket Cannabis Association). Thailand’s Food and Drug Administration (FDA) were considering the idea of a Cannabis Sandbox, which would be similar to Phuket’s Sandbox scheme, but with a focus on cannabis. The Cannabis Sandbox would potentially allow individuals over 20 years of age to use cannabis for recreational purposes and allow tourists participating in the scheme to access cannabis products for treatment in specific areas if they fall ill during their trip. The cannabis sandbox would allow tourists to help create destinations synonymous with the drug such as The Netherlands, according to an interview with Belaws^4^ and The President of Phuket Cannabis Association. They wanted to explore regulations where cannabis would be prohibited for people under 20 years of age, pregnant or lactating women and in areas outside the “RS” zone. In line with the definition for RS as per (Rinnge and Rouf 2020; Ranchordas 2021a; Leimüller and Wasserbacher-Schwarzer 2020; EIPA 2021; https://www.europarl.europa.eu/RegData/docs_autres_institutions/commission_europeenne/com/2021/0206/COM_COM(2021)0206_EN), this intention would have fit with an RS tackling specific clientele during the specific time of Covid and post covid as a designed testbed for the roll out.

However, the government resorted to rapid decriminalization of cannabis into the country to attract tourism and rule out a lengthy RS process (Belaws and an interview with the CEO of AgriTech and R2 Holdings operating in Medical Cannabis in Thailand). As of early 2024, Thailand’s new government was set to pass legislation banning cannabis for recreational use, 18 months after the country became the first in Asia to decriminalize the plant. This announcement was followed by a back and forth of recrimination vs. decriminalization of Cannabis causing a state of confusion and chaos in the industry. The relaxed laws led to a cannabis industry serving both locals and foreigners and hence not attaining its initial objective.

Since then, Thailand has found itself at the centre of a complex debate about how to regulate Cannabis. The government and public has been struggling to strike a balance between public health concerns, economic opportunities, and changing social attitudes toward cannabis. In September 2024, the government announced a new legislative proposal was under consideration in relation to the Cannabis Laws. This proposal could reshape how cannabis is used and commercialized in Thailand (https://belaws.com/thailand/new-cannabis-laws-in-thailand/).

Looking at the Pros and Cons of this country’s experience, and even though the large decriminalization law allowed a surge of economic gains to be benefitted, investors and business owners have been struggling to understand the legal stance of the country and debating whether they close or move their capital somewhere more stable. Mainly, it caused business owners in Thailand to lose trust with governmental legislation that was considered not thought through. Moreover, the prevalence of cannabis everywhere, has been challenging for minors and their parents.

## Results and analysis

Thomann and Maggetti (2017) explain that the QCA technique entails systematic data analysis, such as truth table analysis and logical minimization, which are conducted based on observations in the dataset (Rihoux and Ragin 2009).

Table 3 Summarizes the three case studies, and the conditions interpreted for each.

**Table 3:** Summary of case conditions

	New Cannabis Product/ Service	New Policy	RS Implementation	Outcome
Brazil	Medical Cannabis Products	Setting guidelines for the production and distribution of Cannabidiol-based medications	An RS was implemented and monitored for a small sample.	Solved the problem for ill people to receive cannabis and paved the way for an incremental legalization.
Arizona	Payment service for Cannabis Businesses	Set the standard for financial technology solutions geared to the cannabis sector’s specific demands	An RS was implemented and monitored for a group of cannabis businesses in Arizona.	Promoted economic growth while assuring adherence to regulations.
Thailand	Recreational Cannabis	Setting regulations to allow individuals over 20 years of age to use cannabis for recreational purposes and allow tourists to access cannabis products	An RS implementation was scrapped out at the last minute and a full legalization was enacted.	Reported boosted tourism in an unstable regulatory framework.

Source: Authors’ accumulation of case data

The outcome is measured based on the success criteria of why the RS was implemented or intended to be implemented in the first place. The success criteria in Brazil for example was enabling terminally ill patients to have access to cannabis easily in a regulated environment. On the other hand, the success criteria in Arizona were regulating cannabis payments without the legal and banking hurdles and in line with state laws. Finally in the case of Thailand, the success criteria as per the interviews were to boost tourism in a controlled and regulated way.

The truth table below (Table 4) was constructed to explore which various combinations of conditions, including new industry, new cannabis product/service, new policy and RS implementation were enough to determine whether the outcome was successful in introducing the Cannabis product/ service in Brazil, Arizona, and Thailand. The conditions were extracted from the case studies section based on the conditions that the nation decided to implement or not the RS.

**Table 4:** Truth table

New Industry (NI)	New Cannabis product/ service (NC)	New policy (NP)	RS Implementation (RI)	Outcome	Case
1	1	1	1	1	Brazil
1	1	1	1	1	Arizona
1	1	1	0	0	Thailand

Source: Authors’ extrapolation of case study data

QCA scoring is based on binary scores and referred to as ‘crisp set’ QCA. This method involves assessing each condition or variable as either present (1) or absent (0), leading to a binary classification of cases. Crisp-set QCA is commonly used when conditions are clearly defined and there is no ambiguity in their classification.

For example, as shown in Table 4, in the cases of Brazil and Arizona, all necessary conditions for RS success—a new industry, a new cannabis product/service, a new policy, and RS implementation—are met (all values = 1), resulting in a successful RS outcome (Outcome = 1). Conversely, in Thailand, the absence of the ‘Implementation of RS’ condition (value = 0) led to the failure of the Success Criteria (Outcome = 0), despite the presence of the other conditions.

NI*NC*NP*RI → Policy was successful in introducing the Cannabis product.

NI*NC*NP ~ RI → Policy was not successful in introducing the Cannabis product.

A necessary condition is required for a given outcome to occur, but it is not sufficient to ensure the occurrence (Thomann 2020). Through our analysis, “Implementation of RS” emerges as a necessary factor for the success of outcome in the cannabis sector.

When deriving the sufficient condition/s in our study, the logical minimization process showed that only the “AND” method is applicable, whereby all four conditions are deemed sufficient as they must be simultaneously present for the desired outcome to occur.

In this context, the success of RS for cannabis use was exemplified in Brazil and Arizona. Notably, the RS in Arizona exhibited marked success, stimulating economic advancement, and enhancing operational safety measures within the region. As in Brazil, the RS framework fostered notable strides in the realm of medical manufacturing, catalyzing innovative breakthroughs. Moreover, it facilitated more streamlined and effective rule-making processes, along with efficient resource allocation practices. However, the lack of meeting all the requisite conditions for RS implementation in Thailand underscores the desired outcome of the legalization. The Thai government could have greatly benefited from a RS when it was trying to determine how to legalize cannabis. Like The Netherlands that adopted fast legalization before fine-tuning the legal and administrative details, the administrative toleration became largely regarded as a failure to legalize.

## Conclusions and policy implications

This paper has assessed the effectiveness of RSs implementation in the cannabis industry across three countries. The analysis revealed promising outcomes in countries where RS was successfully implemented, demonstrating its potential to stimulate innovation, generate revenue and bolster regulatory supervision within the cannabis industry.

Specifically, our findings illustrate that the establishment of efficient regulatory frameworks can foster the industry’s growth and development. Accordingly, we propose the introduction of a RS in Germany tailored to accommodate cannabis edibles since edibles have not been addressed yet in the anticipated legalization plan in Germany in 2024. However, this theory has only been approached from an innovation and economic perspective and requires an in-depth legal and socio-economic analysis.

Our Review of the EU RS and the German Reallabore reveals substantial differences between both frameworks in terms of scope, implementation and regulatory flexibility. While the EU framework is more expansive and flexible, enabling a range of industries and experimental methodologies, the German approach is often more focused and aimed towards certain sectors with clear legal trials. This implies that Germany would benefit from implementing a more accommodating and inclusive regulatory structure, similar to the EU’s, particularly in supporting developing sectors like cannabis edibles. Additionally, strengthening collaboration between German and EU regulators could enhance these frameworks’ effectiveness, ensuring they support innovation while maintaining necessary oversight.

Although the focus of our research is cannabis as a whole, with particular attention given to regulatory mechanisms, it is crucial to acknowledge the growing market for consumable cannabis products in Germany. Whether medical or recreational, cannabis edibles as a form of cannabis or method of consumption needs to be carefully strategized and explored.

Our results of performing a QCA analysis on three cases where RS were used or planned to be used, demonstrated the importance of the efficacy of RS implementation in cannabis along with the introduction of a new policy or regulation for a new product or service in a new industry. Whether the experience of the Brazil and Arizona can be transferred to Germany and tailored to a specific cannabis product requires an in-depth regulatory assessment.

Given that the legalization and regulation of cannabis edibles offer potential advantages as well as difficulties for governments; by strategically implementing RSs and other policy interventions, regulators may effectively negotiate the complexity of the cannabis business and assure its responsible and sustainable evolution.

Policymakers should prioritize education and awareness campaigns as well as regulatory interventions to adequately inform consumers about the potential benefits and risks associated with cannabis edibles. Moreover, continuous evaluation and research are required to track the effects of regulatory interventions and adjust based on empirical evidence when required.

Finally, to maintain political support for this flexible regulatory instrument while balancing monitoring and enforcement methods within the sandbox, regulators may need to implement additional strategies. For example, strategic collaboration with non-state regulatory systems and third-party actors may make it easier to achieve this equilibrium (Johnson 2023). It is also important to state that not every sandbox experiment can be carried out legally or smoothly or reach the desired outcomes of its founding.

Prior to implementing a sandbox tool, it is advisable for analysts to examine sector-specific considerations and determine the ways in which sandboxes can facilitate innovation that is both socially responsible and responsive (Stilgoe et al. 2013). When formulating sandboxes for other sectors, policymakers must exercise caution to avoid conflating financial risks with those pertaining to human health, the environment, or social justice. (Johnson 2023).

## Data Availability

I herewith formally declare that the authors of this (“Manuscript”) have written the submitted dissertation independently. We did not use any outside support except for the quoted literature, databases and other sources mentioned in the paper. I clearly marked and separately listed all the literature and all of the other sources which I employed when producing this academic work, either literally or in content.

## Footnotes

1
https://www.bmwk.de/Redaktion/DE/Textsammlungen/Digitale-Welt/reallabore-konsultation.html. Real Laboratories (Regulatory sandboxes) make it possible to test innovations that encounter limitations or open questions in the general legal framework for a limited period of time under conditions that are as realistic as possible and with regulatory oversight. In many cases, real-world laboratories are based on experimental clauses that enable the responsible authority to allow controlled exceptions to technical legal requirements and prohibitions for testing purposes.

2
https://www.cdc.gov/cannabis/about/state-medical-cannabis-laws.html.

3 RSs recognized as specialized platforms that allow organizations to test new products, services or business models under regulatory supervision for a limited time. Their primary goals are to foster business innovation through real-world testing and to aid in developing legal frameworks that support these activities (Rinnge and Rouf 2020; Ranchordas 2021a; Leimüller and Wasserbacher-Schwarzer 2020; EIPA 2021; https://www.europarl.europa.eu/RegData/docs_autres_institutions/commission_europeenne/com/2021/0206/COM_COM(2021)0206_EN).

4 Belaws is a one-stop service designed to assist entrepreneurs and corporations managing their legal, corporate and accounting matters in Thailand.
